# Global Ethical Principles in Healthcare Networks, Including Debates on Euthanasia and Abortion

**DOI:** 10.7759/cureus.59116

**Published:** 2024-04-26

**Authors:** Safa Mousavi

**Affiliations:** 1 Public Health, California State University, Fresno, USA

**Keywords:** principle of ethics, abortion, euthanasia, nonmaleficence, beneficence, justice, autonomy

## Abstract

In today's ever-evolving healthcare landscape, the focus is shifting towards integrated care, inter-organizational cooperation, and healthcare networks (HCNs) as alternatives to traditional healthcare institutions. This transformation is driven by factors such as an aging population and increasing healthcare costs, necessitating a reevaluation of ethical considerations to ensure the well-being of patients remains central. This review provides a narrative overview of ethics within HCNs, with a focus on patient-centered medical ethics. It elaborates on the four fundamental ethical principles, namely justice, beneficence, nonmaleficence, and autonomy. The principle of justice underscores HCNs' ethical obligation to provide equitable and transparent access to all patients, ensuring fairness in resource allocation. The principle of nonmaleficence underscores the responsibility to prioritize patient safety, while beneficence obliges HCNs to ensure continuity of care across all dimensions. Furthermore, the principle of autonomy is redefined as a commitment to actively promote and respect patient choices. HCNs that do not adhere to these ethical principles raise concerns and lack ethical justification. Additionally, the review delves into the legal aspects of euthanasia and abortion, both of which present complex ethical challenges in healthcare systems globally. A comparative analysis is provided, examining their legal status in Islamic countries, European nations, and the United States. This study sheds light on the evolving ethical landscape in HCNs and the diverse global perspectives on contentious issues. Therefore, harmonizing legislation and defining appropriate boundaries are crucial steps toward upholding ethical standards in healthcare practices on a global scale.

## Introduction and background

Healthcare organizations are stepping towards a new generation. Integrated care, inter-organizational cooperation, and healthcare networks (HCNs) are rapidly becoming the focal points of attention in the delivery of medical treatment, replacing the separate healthcare institutions that have historically served as the sector's primary support structure [1]. These shifts are the consequence of a number of different causes, including an aging population and the rising costs of the welfare system [1], which have led to a decrease in the availability of medical services [1]. There are high expectations for these types of partnerships and HCNs, which, according to the findings of certain research, have the potential to boost economic efficiency [2] and ultimately result in an improvement in the quality of medical treatment [3]. However, improving the quality of treatment does not provide ethical justification.

When discussing ethical duties for networks, one of the most prevalent approaches is to examine the problem from the point of view of the ethics of organizations or businesses. This makes sense since the question is about how organizations like HCNs should be set up. Following this comes the question of how the legal and moral responsibilities should be dispersed across the vast network. However, studies demonstrate that patients don't behave as equal or rational consumers within the healthcare sector [4]. Many individuals seeking medical services don't assess different healthcare organizations or networks in terms of service quality and costs [1]. These factors underscore the potential dangers of assuming that healthcare organizations function primarily as businesses. In addition, there is a possibility that adopting a business ethics strategy may result in a devaluation of the pivotal role that patients play in the healthcare system since this strategy will consider patients to be only one stakeholder among many others. It is possible that as a result of this, the interests of patients will be given less weight than those of other stakeholders [1].

However, the fundamental concept of medical practice is predicated on the ethical imperative of placing the patient's well-being ahead of all other concerns. Thus, it would be more beneficial to establish an ethical approach to HCNs that begins with patients and the ethical obligations they might make on these networks [1]. Enshrined in medical ethics rules all around the globe is the responsibility that healthcare professionals have to put their patients' needs ahead of their own, which is often considered to be the most important ethical commitment they have [5].

Ethical requirements for HCNs provide a challenge, but it is feasible to establish specific ethical duties by using the traditional four principles of medical ethics: autonomy, justice, beneficence, and nonmaleficence [1]. Therefore, when faced with moral dilemmas on the job, medical professionals and other healthcare workers may refer to these principles for guidance in reaching a decision. The principles of medical ethics serve as a framework for the interactions that doctors have with their patients, their colleagues, and society in general. It provides behavioral and decision-making standards that assist physicians in understanding what is expected of them by their colleagues, their patients, and society in general. Also, the ethical rules of the World Medical Association (WMA) serve as the foundation for deciding what constitutes suitable behavior on the part of physicians with regard to these issues [5].

One strategy to follow in HCNs is to make medical ethics an explicit focus of attention in the physician-patient relationships. These connections often contain ethical conflicts between two or more interests, which doctors are obligated to notice and figure out how to address. In addition to this, it throws light on key social issues that are relevant to the practice of medicine such as euthanasia, abortion, organ transplantation, and end-of-life (EOL) medical research [6]. This review outlines and examines ethical issues in HCNs' ethical obligations toward individual patients and medical practice in general, with a specific focus on those issues that are faced by physicians in their relationships with their patients.

Method

A narrative literature review was conducted using PubMed and Google Scholar databases, focusing on English sources published after 2013 that addressed principles of ethics and their challenges in HCNs. The rationale behind selecting articles from the last decade was to ensure access to the most recent and updated information pertaining to ethical considerations and contextual issues in HCNs. The search terms employed included Principle of Ethics, Autonomy, Justice, Beneficence, Nonmaleficence, Euthanasia, and Abortion. Inclusion criteria encompassed articles discussing these principles and their application in HCNs, as well as debates surrounding euthanasia and abortion within this context. Following the search, articles were screened and sorted based on relevance to the research objectives. Subsequently, the necessary data was extracted and independently evaluated for reliability. This systematic approach aimed to provide a comprehensive understanding of ethical principles and challenges in HCNs, along with debates on contentious issues like euthanasia and abortion.

## Review

The patient and the four principles

**Figure 1:**
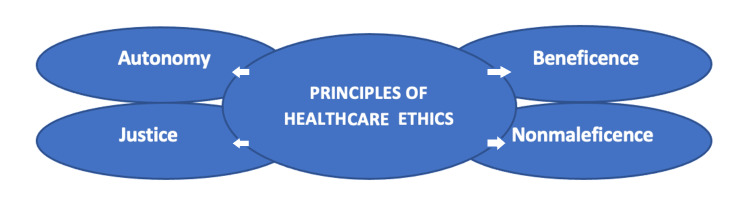
The four principles of healthcare tthics

Patients have the ability to make substantial ethical claims on the organizations and networks that provide healthcare. In clinical treatment, patients can anticipate that HCNs will fulfill their ethical duties of autonomy, justice, beneficence, and nonmaleficence (Figure 1). This is a common method for classifying these ethical claims, and it is based on the well-known and widely accepted principles of Beauchamp and Childress [7]. While this framework is often used to discuss the ethics of patient-physician relationships in clinical care, I believe this responsibility applies to HCNs as well. In clinical care, practitioners of healthcare have ethical obligations toward particular patients. However, in HCNs, these duties take the shape of more generic "duties to design" [8]. This comprises requirements to construct the network in such a way that the interests of all patients (both existing and prospective) inside the network are preserved.

HCNs' ethical duties toward patients

Justice and Access to the Network

Justice is sometimes described as the fair, equitable, and right treatment of persons. Of the numerous varieties of justice, distributive justice is the most important to clinical ethics. In the context of healthcare, "distributive justice" relates to the fair, equitable, and adequate allocation of healthcare resources. This distribution is established by justifiable norms that frame the conditions of social cooperation [9]. It is generally accepted that every patient has an ethical right to access healthcare that is fair and equal [10]. The subject of justice is especially crucial for HCNs since they have the ability to greatly influence how healthcare is structured and provided within a healthcare system.

It is an undeniable fact that the resources of HCNs are limited, and it is necessary to choose a principle of distributive justice that is reasonable for distributing these restricted resources. If access is refused or limited, it should be done for legitimate reasons that are transparent, known to and understood by the patient. So, patients have a legal right to be informed about the process by which access to a network is governed, as well as the guiding concepts that underlie this process. Access to healthcare is determined by a number of factors, including socioeconomic status and geographical location. Numerous studies have shown that those with better socioeconomic status have easier, quicker, and more convenient access to medical treatment. Socioeconomic status is a major contributor to healthcare disparities in the United States [11]. In terms of geography, the choice of an HCN to provide a certain service at a single site may benefit people who have the physical and financial ability to go to this area. Healthcare service must ensure that such a strategy does not interfere unnecessarily with a patient's ability to choose their own healthcare provider and, more significantly, does not unjustly favor individuals who live near the network hospitals delivering a specific care. It is important to organize the delivery of certain medical services inside that network to ensure that all potential patients within the network's coverage have access [1].

Nonmaleficence and Safety

Nonmaleficence is a cornerstone of medical ethics. The famous "first do no harm" is often equated to this principle. The idea of injury, on the other hand, is still up for dispute, as both Beauchamp and Childress pointed out in their analysis of this principle. The terms "wronging" and "hurting" are occasionally distinguished from one another. Wronging is thought to be a violation of one's legal or moral rights while harming is understood to be a setback to one's interests [7]. As a result, one may be treated unfairly without really suffering any physical injury. For instance, if a patient's private medical data are shared with third parties who do not have justified access to these data, the patient has been wronged. This is the case even if the patient was uninformed of this sharing and experienced no loss of interest as a consequence. We regard the nonmaleficence principle to be wider than the "first-do-no-harm" concept and to include the obligation not to mislead patients.

Nonmaleficence refers to a physician's obligation to avoid inflicting needless suffering on a patient. This idea, which is articulated in a plain way, gives support for a variety of moral rules, some of which are as follows: do not commit murder, do not cause pain or suffering, do not incapacitate, do not offend, and do not deprive others of the joys of life. In the field of medicine, the ethical principle of nonmaleficence is put into practice when a doctor weighs the benefits of a patient's care against the costs of any and all possible interventions and treatments, avoids those that are unnecessarily burdensome, and decides which course of treatment will be most beneficial to the patient by considering all of the options available to them [9].

Beneficence and Continuity of Care

Patients also have the right to make the valid argument that HCNs are structured so as to be of the greatest possible benefit to them. First, this indicates that HCNs have to be justified in terms of the degree to which they either give more care or enhance the quality of care already provided. It is often assumed that HCNs may increase quality, such as by increasing the likelihood of delivering seamless and integrated treatment in an environment in which medical professionals from a variety of specialties collaborate. Secondly, when patients become part of a healthcare network (HCN), they will undergo a specific path of care, which may involve specific healthcare providers within the network. However, from the patient's point of view, how this path is organized is not usually of significant concern. The main focus for patients is their right to receive high-quality care, and ethically, they also have the right to expect this care to be delivered smoothly and consistently throughout their entire treatment journey within the network [12]. This trajectory's structure is mostly immaterial from the patient's standpoint. Patients not only have a legal right to get great medical treatment, but they also have an ethical right to receive care that is consistent, effective, and of a high standard throughout the whole of their care journey. Therefore, HCNs need to be constructed in such a way as to optimally assure continuity of care. There are three dimensions involved here, as given below.

Ensure information continuity: HCNs must guarantee patient information is transmitted securely and efficiently. The HCN must find a balance between disclosing too much (which might injure the patient and violate the duty of nonmaleficence) and too little (which fails to maximally benefit the patient and breaches the duty of beneficence).

Ensure managerial continuity: A network should have a common strategy for managing a health issue. Patients may be harmed if transported inside a network and treated differently each time.

Ensure relational continuity: Relationships between patients and physicians continue to be the foundation of contemporary medical practice.

Patient Autonomy

Immanuel Kant (1724-1804) and John Stuart Mill (1806-1873) viewed autonomy as an ethical concept based on the idea that all people have inherent and unconditional value and should be able to make logical judgments and moral choices and use their ability for self-determination [13]. In 1914, Justice Cardozo wrote a ruling in which he supported this ethical concept by saying, "Every human being of mature years and sound mind has a right to select what will be done with his own body" [9].

Autonomy encompasses individual privacy, freedom of choice, self-control, and the ability to make one's own well-informed judgments. In accordance with the ethical concept of autonomy, HCNs have the moral imperative to both respect and actively promote patient autonomy in their practice. This principle could involve the freedom to make as many autonomous decisions as possible about one's treatment trajectory, as well as the right to choose one's chosen healthcare expert or provider. HCNs should become maximally efficient while protecting patient autonomy. Thus, it is conceivable for HCNs to create a network in such a way that patients have a greater chance of being matched with caregivers who deliver the highest quality of care that is consistent with the patients' individual values [1]. The autonomy principle does not apply to those who lack the ability to act independently. This includes infants, children, and individuals with developmental, mental, or physical disorders. Healthcare organizations and state governments in the United States have rules and processes to judge incompetence. When the principle of autonomy is respected, the physician is obligated to provide the patient with the medical information and treatment options that are required for the patient to engage in self-determination. Respecting the concept of autonomy also supports informed consent, confidentiality, and truth-telling [9].

Each of the four ethical standards must be followed unless they conflict with one another. The physician must assess the real responsibility to the patient by weighing the conflicting prima facie responsibilities based on content and context. This ethical obligation to promote autonomy may conflict with HCNs' beneficence duty in specific cases. For example, an HCN may assign a given healthcare service to one network member, sending all patients in need to that institution. All patients at one facility may benefit; however, if networks are constructed such that patients or their data are automatically moved to certain institutions or placed on a particular treatment route, this might contradict the ethical concept of patient autonomy, since patients would be unable to choose where and how they are treated [1]. Furthermore, physicians face many complicated and challenging problems. For example, when a patient in shock treated with an intravenous catheter goes through discomfort, fluid resuscitation, and edema. Beneficence overcomes nonmaleficence. Consider a patient's denial of a life-saving intervention such as mechanical ventilation or desire for a life-ending measure like withdrawing mechanical ventilation. When there is a conflict between autonomy and beneficence, ethical decision-making is most challenging [9].

Medical ethics

**Figure 2:**
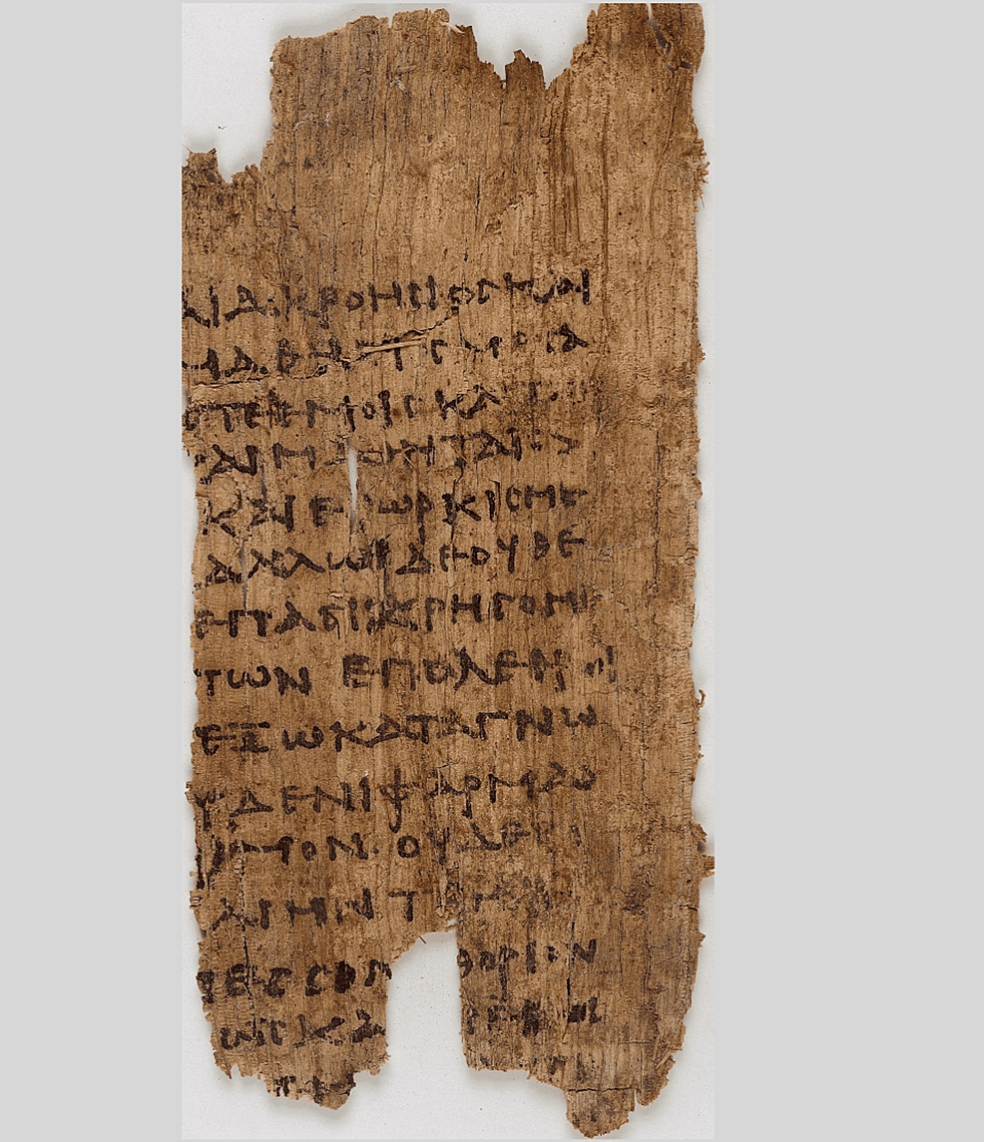
A fragment of the Hippocratic oath on the 3rd-century Papyrus Oxyrhynchus 2547 Image Source: This file comes from Wellcome Images, a website operated by Wellcome Trust, a global charitable foundation based in the United Kingdom. This file is licensed under the Creative Commons Attribution 4.0 International license.

Medicine dates back thousands of years and has undergone gradual change over that time. The earliest mentions of it come from ancient Egyptian and Oriental civilizations, but beyond that, we have very little information about its early past. Hippocrates was the first person to distinguish medicine (as a science) from philosophical notions and magic, both of which were often applied in patient care during his historical period. In other words, Hippocrates was the first person to distinguish medicine as a science. Despite this, a good number of these guiding principles are being practiced today after 2500 years. Hippocratic medicine is like a social contract in that the code of ethics creates a set of standards to be followed by physicians. As a synthesis of this contract, the Hippocratic Oath was created in ancient Greece, and Herodotus and maybe Homer contributed to the composition [14]. A fragment of the Hippocratic oath on the 3rd-century Papyrus is shown in Figure 2. Even though it has been updated in various ways, the present version of the Oath still upholds the same ethical principles.

The original Hippocratic Oath translated into English [14] is given below.

I swear by Apollo the physician, and Aesculapius the surgeon, likewise Hygeia and Panacea, and call all the gods and goddesses to witness, that I will observe and keep this underwritten oath, to the utmost of my power and judgment.

I will reverence my master who taught me the art. Equally with my parents, will I allow him things necessary for his support, and will consider his sons as brothers. I will teach them my art without reward or agreement; and I will impart all my acquirement, instructions, and whatever I know, to my master’s children, as to my own; and likewise, to all my pupils, who shall bind and tie themselves by a professional oath, but to none else.

With regard to healing the sick, I will devise and order for them the best diet, according to my judgment and means; and I will take care that they suffer no hurt or damage.

Nor shall any man’s entreaty prevail upon me to administer poison to anyone; neither will I counsel any man to do so. Moreover, I will give no sort of medicine to any pregnant woman, with a view to destroy the child.

Further, I will comport myself and use my knowledge in a godly manner.

I will not cut for the stone, but will commit that affair entirely to the surgeons.

Whatsoever house I may enter, my visit shall be for the convenience and advantage of the patient; and I will willingly refrain from doing any injury or wrong from falsehood, and (in an especial manner) from acts of an amorous nature, whatever may be the rank of those who it may be my duty to cure, whether mistress or servant, bond or free.

Whatever, in the course of my practice, I may see or hear (even when not invited), whatever I may happen to obtain knowledge of, if it be not proper to repeat it, I will keep sacred and secret within my own breast.

If I faithfully observe this oath, may I thrive and prosper in my fortune and profession, and live in the estimation of posterity; or on breach thereof, may the reverse be my fate! [12]

Ethics has always been an essential part of the medical profession, regardless of historical period or country. The principles of medical ethics guide the interactions that physicians have with their patients, their colleagues, and society in general. As a result of the fact that ethics are founded on philosophies, religions, and political ideologies, there are significant differences in the medical ethics that are practiced in different countries [6]. Both euthanasia and abortion are two of the most controversial topics in ethics, medicine, and the law that have dominated the 21st century and among religions. These topics have sharply divided the scientific and nonscientific public into advocates and opponents of each issue. Here, a brief comparison of their legal status and medical ethics in Islamic countries, European countries, and the United States is provided.

Euthanasia

Euthanasia is the practice of intentionally ending life to eliminate pain and suffering. It is widely seen as a humanitarian approach to terminal prognosis and patient pain. Emotional arguments, usually about severe circumstances, challenge the morality against physicians taking human life prematurely. This ethical concern has not only involved physicians but has also captivated legal and sociological experts worldwide throughout history. Legislators generally align with one of three approaches: outright prohibition of euthanasia, equating it with ordinary or privileged murder, or permitting it under certain prescribed conditions [9].

In Islamic countries, euthanasia is considered to be equivalent to murder. Thus, it is outlawed in all nations that are governed by Islamic religious beliefs. Iran does not make an exception. In other Islamic countries like Turkey and a portion of Bosnia and Herzegovina, euthanasia is seen in the same light as other forms of murder and is subject to the same severe penalties [15]. However, the Benelux nations (Netherlands, Belgium, and Luxembourg) are Western European nations that do not consider the taking of life from people to be a crime if it is done in accordance with established legal guidelines and medical protocol. In the Netherlands, euthanasia could be requested not just by competent adults, but also by youth above the age of 12 [16]. While euthanasia is illegal in the United States, 10 states and Washignton, DC, have legalized physician-assisted suicide [17]. In this approach, we demonstrate how a similar scenario in real life might be governed quite differently by various legal systems.

Abortion

Abortion is one of the most commonly debated medical ethics topics in the world. Induced abortion is a practice that may be found in all nations, but the choice to terminate a pregnancy must take into account a wide range of factors, including those pertaining to medicine, ethics, morality, religion, society, the economy, and the legal system.

There are differing perspectives on abortion in Islamic ethics. Whether the fetus is believed to be alive is the first source of disagreement. It is believed by some sources that ensoulment occurs about 120 days after conception. Ensoulment is considered to be the first tangible evidence of life. This suggests that it is illegal to get an abortion beyond the first 120 days of a pregnancy. However, it is not plausible to assert that Islam permits abortions prior to the 120 days of pregnancy. Turkey is the only Muslim nation with secular democracy and legal abortion [18]. In addition, the laws of each state in the United States have drastically different policies regarding the legality of abortion as well as the many limits that are placed on the operation. However, today almost all European countries allow abortion on request [19]. Thus, the investigation has shown that the agreement law addressing abortion is more complex than could be expected.

This review's strength lies in its meticulous approach to exploring ethical principles and challenges in HCNs through a study of literature from reputable databases. However, a potential limitation arises from its exclusion of countries like China and India, along with other oriental nations, which may limit the global applicability of the findings. Furthermore, the absence of a discussion on racial discrimination within HCNs could limit the depth of the analysis.

## Conclusions

In response to significant obstacles, the healthcare system is undergoing a fast transformation. At the moment, a lot of people are placing their hopes on comprehensive HCNs and integrated medical services. The empirical implications of such networks are being discussed but it is important that a greater focus be given to the ethical considerations involved. We are in need of a framework that will allow us to evaluate how and when HCNs are justifiable so that we can address the new and serious ethical challenges that they generate. Furthermore, how to place medical ethics more firmly into medical practice continues to be a central concern of physician training and practice in healthcare systems. This review also shows how a life situation may be regulated differently in different legal areas and this is another challenge faced by HCNs.
